# Supplementary figures and images for: The Composite Severity Score for Lumbar Spine MRI: a Metric of Cumulative Degenerative Disease Predicts Time Spent on Interpretation and Reporting
Source: J Digit Imaging. 2021 May 23;34(4):811–9. doi: 10.1007/s10278-021-00462-1 (PMC8455764; doi:10.1007/s10278-021-00462-1)

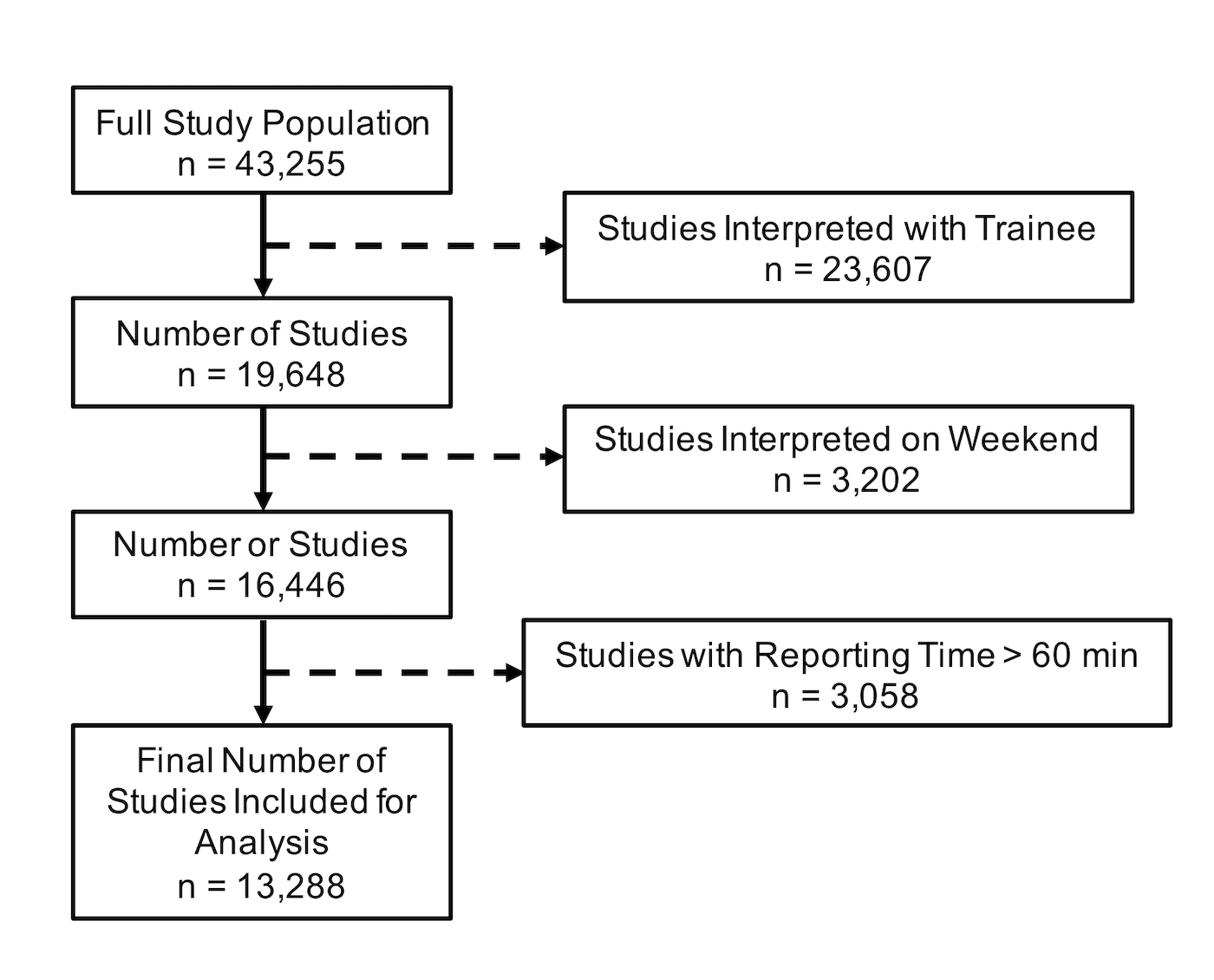

Supplement: Supplementary file 1 — Supplementary file1 (TIFF 6181 KB) [file 10278_2021_462_MOESM1_ESM.tiff]

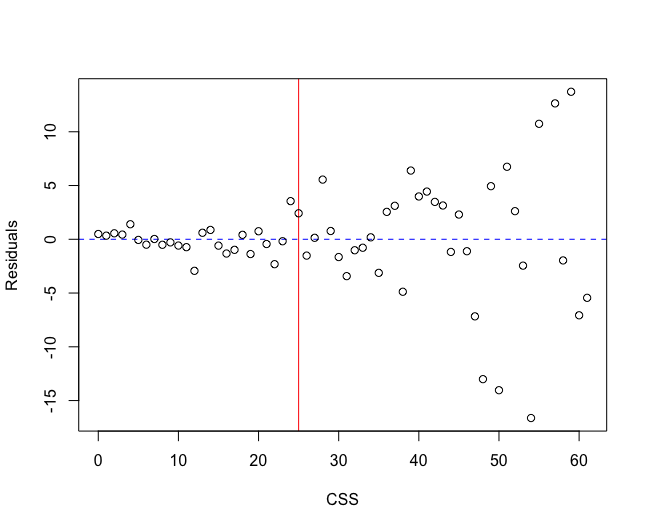

Supplement: Supplementary file 2 — Supplementary file2 (TIFF 1339 KB) [file 10278_2021_462_MOESM2_ESM.tiff]

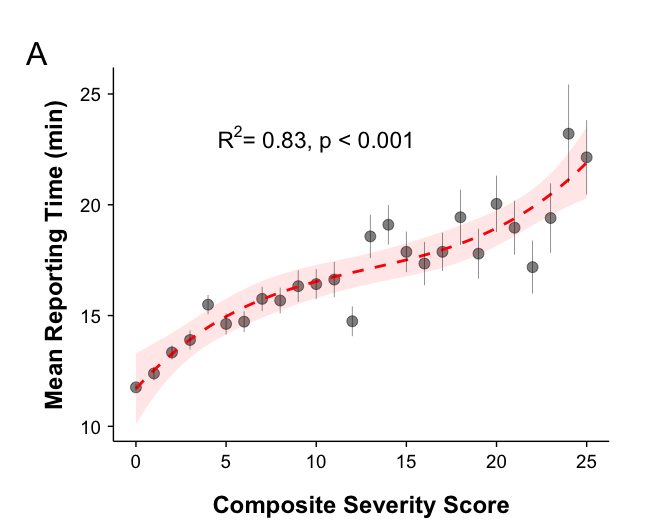

Supplement: Supplementary file 3 — Supplementary file3 (TIFF 1339 KB) [file 10278_2021_462_MOESM3_ESM.tiff]

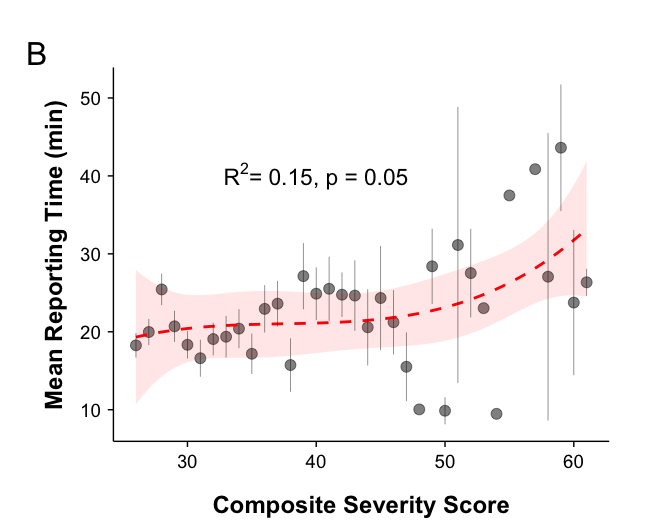

Supplement: Supplementary file 4 — Supplementary file4 (TIFF 1339 KB) [file 10278_2021_462_MOESM4_ESM.tiff]
