# Supplementary material for: The Composite Severity Score for Lumbar Spine MRI: a Metric of Cumulative Degenerative Disease Predicts Time Spent on Interpretation and Reporting
Source: J Digit Imaging. 2021 May 23;34(4):811–9. doi: 10.1007/s10278-021-00462-1 (PMC8455764; doi:10.1007/s10278-021-00462-1)
Supplement: Supplementary file 5 — Supplementary file5 (DOCX 76 KB) [file 10278_2021_462_MOESM5_ESM.docx]

Title:

*The Composite Severity Score for Lumbar Spine MRI: A Metric of Cumulative Degenerative Disease Predicts Time Spent on Interpretation and Reporting*

**Abstract:**

*Background and Purpose:*

Conventional measures of radiologist efficiency, such as the *relative value unit*, fail to account for variations in the complexity and difficulty of a given study. For lumbar spine MRI (LMRI), an ideal performance metric should account for the global severity of lumbar degenerative disease (LSDD) which may influence reporting time (RT), thereby affecting clinical productivity. This study aims to derive a global LSDD metric and estimate its effect on RT.

*Materials and Methods:*

A 10-year archive of LMRI reports comprising 13,388 exams was reviewed. Objective reporting timestamps were used to calculate RT. A natural language processing (NLP) tool was used to extract radiologist-assigned stenosis severity using a 6-point scale (0 = “Normal to 5 = “Severe”) at each lumbar level. The composite severity score (CSS) was calculated as the sum of each of 18 stenosis grades. The predictive value of CSS, sex, age, radiologist identity, and referring service on RT were examined with multiple regression models.

*Results:*

The NLP tool accurately classified LSDD in 94.8% of cases in a validation set. The CSS increased with patient age and differed between men and women. In a univariable model, CSS was a significant predictor of mean RT (R^2^ = 0.38, p <0.001) and independent predictor of mean RT (p <0.001) controlling for patient sex, patient age, service location, and interpreting radiologist. The predictive strength of CSS was stronger for the low CSS range (CSS = 0-25, R^2^ = 0.83, p <0.001) compared to higher CSS values (CSS > 25, R^2^ = 0.15, p = 0.05). Individual radiologist study volume was negatively correlated with mean RT (Pearson’s R = -0.35, p <0.001).

*Conclusion*:

The composite severity score predicts radiologist reporting efficiency in LMRI, providing a quantitative measure of case complexity which may be useful for workflow planning and performance evaluation.

**Introduction:**

Efficient interpretation and reporting are central principles of delivering value in diagnostic radiology.^1^ Common performance metrics such as the relative value unit (RVU) are used to quantify the value of physician work as a function of time^2^. However, the RVU does not account for variability in procedural complexity which may significantly alter the duration of a medical procedure^3^. This discrepancy can generate systemic bias and misaligned incentives for physicians and healthcare organizations^4^. In diagnostic radiology, we hypothesize that studies with more complex pathology would require more cognitive energy, resulting in longer reporting time (RT) that is not captured by the RVU.^5–7^

To address this question, we studied degenerative disease of the lumbar spine (LSDD) on MRI (LMRI). Compared with other imaging reports, LMRI reporting is typically structured by anatomic level and each level is assigned a grade of relative severity of stenosis.^9^ Prior work has shown that inter-radiologist agreement for spinal canal stenosis (SCS) and neural foraminal stenosis (NFS) is moderate to strong. ^1–5^ Moreover, the descriptive terminology for LSDD is standardized by an interdisciplinary consortium of national societies allowing comparison between radiologists with different training and levels of experience.^14^ These features enable analysis of reporting text by natural language processing (NLP) algorithms, which allow a large corpus of text to be analyzed rapidly.

We hypothesized that a measurable relationship exists between disease severity (complexity) and RT owing to increased cognitive energy required to render a diagnosis and assign a severity value.^15,16^ The primary aim of this study was to quantify the relationship of radiologist-assigned LSDD severity and reporting time, leveraging NLP to analyze a large volume of reports from many individual radiologists. To this end, we present and validate an NLP tool and derive a composite “severity score” reflecting the cumulative severity of LSDD at 18 discrete sites in the lumbar spine. We then evaluate the predictive value of this composite severity score on objective reporting times.

**Methods and Materials:**

Data Selection

We performed an institutional review board-approved review of LMRI reporting data in compliance with the Health Insurance Portability and Accountability Act for which informed consent was waived. The 10-year archive of report text and reporting metadata comprised 43,255 LMRI studies from a single, multi-site institution (BLINDED FOR REVIEW), representing 29 different MR scanners. Studies which were co-interpreted with trainee assistance (n = 23,607), reported on the weekend (n = 3,202)were excluded because the workflow for these studies was presumably different. We also excluded studies in which the calculated RT was over 60 minutes (n = 3,058) because this was felt to be beyond the conventional RT for an attending radiologist without significant interruption (exclusion criteria summarized in supplemental Figure 1).^17^ Reports were signed by 152 unique attending radiologists including generalists, and fellowship-trained musculoskeletal radiologists, and neuroradiologists. Patient age, sex, and the requesting service (documented as “Inpatient”, “Outpatient”, “Emergency”, or “Unknown”) were recorded. Reporting documentation timestamps were extracted from the institution’s reporting software API (Powerscribe, Nuance Communications, Burlington VT, USA). The timestamps marked the initialization and finalization of the radiology report to the 1/100 of a second; the difference between these time points was recorded as the RT (min).

Natural Language Processing

We applied a customized NLP algorithm to raw radiology report text. Using a rules-based approach employing a dictionary of customized regular expressions (RegEx), the algorithm was designed to extract a severity rating using a 6 point scale (0 = “Normal”, 1 = “mild”, 2 = “mild to moderate”, 3 = “moderate”, 4 = “moderate to severe”, and 5 = “severe”). A severity score was extracted for spinal canal stenosis (SCS) and left and right neural foraminal stenosis (LNFS, RNFS) for each of six spinal levels: T12-L1 through L5-S1. The NLP was designed using empirically, iteratively developed syntactic and semantic rules including common radiology terminology and phraseology to elicit a severity score (0-5) for the 18 locations (e.g T12-L1 SCS, T12-L1 LNFS), resulting in a 6 x3 matrix for each study (Figure 1). When the model failed to assign a score, a default value of 0 (“Normal”) was applied. To test the accuracy of the model, we randomly selected 100 studies out of the full dataset (n = 43,255) and manually reviewed the radiology reporting text to assess for discrepancy or error. For each case, the reporting text was manually reviewed and assigned a 0-5 value by a radiologist. Scores were considered concordant if NLP and manual review matched exactly and any degree of discordance was considered unsuccessful.

Calculation of severity scores

The composite severity score (CSS) was calculated as the sum of the constituent severity scores (n=18) resulting in scale ranging from 0 (“Normal” at all locations) to a theoretical maximum of 90 (“Severe” at all 18 locations).

The distribution of CSS was assessed for the study population (n = 13,388) and subdivided by age and sex. Age groups were defined as <40, 40-49, 50-59, 60-69, 70-79, and ≥80. The sex-based distributions and age-group distributions were compared to exponential theoretical distributions using quantile-quantile plots. The Kolmogorov-Smirnov (K-S) test was used to test difference in distribution between subgroups.

Quantifying relationships among CSS, Age, Sex, and RT:

CSS and RT were grouped by sex and age and inter-group differences were calculated using ANOVA and pairwise t-tests with the Benjamini-Hochberg method correcting for multiple comparisons. CSS were clustered into deciles and compared to RT using pairwise t-tests. The results are visualized in box-and-whisker diagrams which show the median (central bar), interquartile range (box margins).

Univariate linear least squares regression was used to assess the predictive relationship of CSS, age, sex, service location, and interpreting radiologist on RT. The distribution of residuals indicated heteroscedasticity (Supplemental Figure 2), therefore the regression was performed using log-log transformation. Significant univariate predictors were then grouped, and a multiple least squares regression was performed to assess for independent effects of each variable on RT. For each test, p-value <0.05 was considered significant. A curvilinear regression was then fitted to model predictive value of CSS on mean RT. This was repeated for subgroups of low-CSS (0-25) and high CSS (>25) groups.Unless otherwise stated, values are reported with ± standard error of the mean (SEM). All statistical analysis and data visualizations were performed using R statistical computing software (v 3.6.2, The R Corporation).

**Results:**

Study Characteristics:

From the initial dataset of 43,255 studies, we included 13,388 after applying exclusion criteria (Supplemental Figure 1) representing 12,326 unique patients. The mean patient age was 54.8 ± 0.1 years and the sex distribution was 54.5% women. The mean RT for the study population was 14.41 ± 0.1 min(median = 10.7), with no significant difference in mean RT by patient sex (14.56 ±.15 min for men vs. 14.29±.14 min for women, p = 0.17). These differences are summarized in Table 1.

Natural Language Processing

The NLP accuracy at the level of CSS was 94.8% (93 misclassifications out of 1800 test values in random sample of 100 cases). The NLP was 100% accurate in 5/18 level-instances (27.8%) and was least accurate at right L5-S1, correctly classifying the severity in 86% of cases.

Relationship of CSS, Age, and Sex:

Mean CSS for the full study population was 6.04 ± 0.07. The relationship of CSS and RT to patient age is shown in Figure 2A and showed a moderate positive correlation with Pearson correlation coefficient R = 0.44 (p <.001) and a weak positive correlation between age and RT (Pearson coefficientR = 0.16, p <0.001). The distribution of CSS was exponential and differed by different age group with increasing flattening of the distribution at higher age groups, indicating a shift toward higher CSS (Figure 2B). The distribution of CSS differed between men and women (K-S test, p<0.001, Figure 3A. Mean CSS was higher for men than women overall (7.0 ±0.11 vs. 5.23± 0.09, p <0.001) and the relative difference in proportion by sex is shown for each CSS value in Figure 3B. . The relationships of age and sex to CSS are summarized in Table 1.

Predictive value of CSS on RT

There was a modest, positive correlation between CSS and RT (Pearson’s R = 0.23, p <0.001). In a univariate log-log linear regression model, CSS was a significant predictor of RT (adjusted R^2^ = 0.063) according to the function ln(RT) = 2.14 + 0.17(ln(CSS)) (F_1,13386)_ = 905.6, p <0.001) meaning that for every 1% increase in CSS, we predict a 0.17% increase in RT. The predictive relationship of mean RT to CSS was better modeled using a 3^rd^ order polynomial regression which shows an adjusted R^2^ = 0.38 (p <0.001) (Figure 4) . Polynomial regression for the low CSS range (0-25) showed a stronger predictive value ( R^2^ = 0.83, p <0.001) compared to high CSS range ( >25) (R^2^ = 0.15, p =0.05) (Supplemental Figure 3).

The interpreting radiologist identity was also a statistically significant univariate predictor for RT, accounting for a larger portion of variance than CSS (adjusted R^2^ = 0.45, F_(151,13236)_ = 73.97, p <0.001). Univariate models also identified smaller, significant predictive value of age (adjusted R^2^ = 0.04, F (_1, 13386)=_ 528.4, p<0.001), sex (adjusted R^2^ = <0.01, F_(1, 13386)_ = 4.47, p = 0.03), and requesting service (adjusted R^2^ = 0.026, F_(3, 13384)_ = 118.7,p <0.001). In the multiple regression model incorporating all 5 predictors (CSS, interpreting radiologist, patient age, patient sex, and requesting service), each variable was a significant and independent predictor of RT (p<0.001 for each). In this 5-variable model, the adjusted R^2^ was 0.52 (F_(216, 13171)_ = 67.56, p <0.001) and the interpreting radiologist was the most important predictor (partitioned R^2^ = 0.43) followed by CSS (partitioned R^2^ = 0.043), age (partitioned R^2^ = 0.025, requesting service (partitioned R^2^ = 0.19), and sex (partitioned R^2^ = 0.00024).

We next examined the CSS in 10 groups, based on relative sample size distribution deciles. The relationship of CSS groups and RT is shown in Figure 5. There were significant stepwise increases between groups (ANOVA, p <.001) and pairwise analysis showed a significant, stepwise for each sequential group except the transition from CSS 21-30 to CSS 31-40 (p = 0.74) and CSS 31-40 to CSS 41-50 (p = 0.13) (Table 2).

Because the effect size of individual radiologist identity was large, the mean RT as a function of mean CSS was plotted for individual radiologists, excluding the 5% upper and lower tail outliers (Figure 6). There was a significant positive relationship between mean CSS and RT by radiologist (Pearson’s R = 0.26, p = 0.002). There was also a significant negative correlation between the radiologist’s study volume (number of studies interpreted by each radiologist over the 10-year period) and mean RT (Figure 7A, Pearson’s R = -0.35, p <0.001). Pairwise t-tests between radiologist volume quintiles showed significant decrease in mean RT between the 3^rd^ and 4^th^ quintiles (Figure 7B) (25.02 ± 5.13 min vs. 20.76 ± 5.30 min, p = 0.02) and the 4^th^ and 5^th^ quintiles (20.76 ± 5.30 min vs. 15.00 ± 3.67 min, p = 0.004). There were no significant pairwise differences in the 1^st^-3^rd^ quintiles (p >0.05). The correlation between individual radiologist volume and CSS was nonsignificant (p = 0.75).

**Discussion:**

The CSS is a tool for quantifying radiologic complexity in lumbar spine MRI and is a significant, independent predictor of interpretation and reporting time (RT). The relationship of CSS and RT supports the anecdotal observation that severe LSDD is more challenging to interpret than normal anatomy, measured in terms of time spent generating a final radiology report. The CSS may be useful as a ‘correction factor’, allowing studies to be weighted based on overall complexity. The CSS could then be applied to quality improvement by defining efficiency benchmarks, ensuring equitable distribution of work, and improving existing measures of radiologist productivity (i.e. RVU).

The principle finding of this study is that the CSS is a reasonable predictor of mean RT (R^2^ = 38%). Notably, the CSS performed better at low-range CSS than high-range CSS (R^2^ = 83% vs. 15%), suggesting a de-coupling of this relationship for LMRI studies with large burden of LSDD. In multiple regression, CSS was relatively less important than the identity of the interpreting radiologist. Taken together, these findings indicate that LSDD severity is only one of many factors which influence real-time radiologist efficiency. Nonetheless, we can extract meaningful benchmarks from this large dataset which estimates an increase in median RT of 50% for LMRI studies with a CSS of ~12 relative to a normal study (CSS = 0). The Current Procedural Terminology (CPT) Code for non-contrast LRMI (72148)in the Washington, DC area, is assigned a global RVU of 6.32, of which 1.48 RVU is allocated for physician work (estimated payment ranging from $220.87 to $273.47).^4,19,20^ LMRI can therefore account for a considerable portion of revenue for both academic and private groups. Within a radiology practice, a CSS-weighted RVU could result in more equitable recognition of work, accounting for procedural complexity, a process which has been used to improve performance and quality in pediatrics and surgery^21,22^. Moreover, the CSS was a stronger relative predictor of RT than conventional demographic information (sex, age) and referring service (inpatient, outpatient, emergency). The CSS also remained a robust predictor despite significant variation between radiologists with different training backgrounds and experience level.

Differences observed in CSS by sex and age group are largely consistent with prior cohort studies of LSDD in cohort populations, supporting the feasibility of the model.^23–26^ The distribution of CSS by sex indicate a higher prevalence of normal-low CSS for women and conversely, higher prevalence of high CSS for men, consistent with prior work showing sex-differences in lumbar spine degeneration patterns, attributed to occupational and hormonal differences, gestation, among others. ^27^ In our population, the difference in CSS was significant (7.00 vs. 5.23 for men and women, respectively, p <0.001), and the distribution of CSS also differed by sex. However, despite these anatomic differences, there was no sex difference in RT. It is unclear if this asymmetry is due more to sex-specific differences in natural history and disease prevalence, other unaccounted sex differences in anatomy, or potentially due to implicit bias. While the CSS is not sufficient to directly inform surgical decision-making, the utility of CSS in predicting surgical candidacy as well as its correlation with functional status could be evaluated in future work.

Lastly, while CSS was an important predictor of RT, the effect size attributable to different radiologists was 10-fold higher in the multiple regression model (43% vs. 4.3%). In a population of 152 radiologists, significant variation in training, experience, and clinical role could all contribute to this effect. Still, the relationship of CSS and RT showed a significant correlation across radiologists despite substantial differences in individual practice patterns (R =0.26, p = 0.002). There was a highly significant negative relationship between cumulative study volume and RT indicating that radiologists who read more LMRI over the 10-year period tended to report studies more efficiently. This effect was driven largely by the top quintile of radiologists, who read a disproportionately high number of studies. This may reflect experience; radiologists with higher cumulative volume have likely been in practice longer and may need less time to interpret LMRI; alternatively, radiologists who interpret studies quickly (regardless of experience) are likely to have higher volume overall. The impact of CSS on individual reader practice could be studied in future, prospective work.

The major strength of this study is the large and heterogenous dataset from which the input and outcome variables are derived. By modelling the LSDD pattern of over 12,000 unique patients, the CSS reflects large sample distribution which is likely representative of the general population. The CSS also incorporates the interpretation styles and practice behaviors of over 150 radiologists. The use of timestamp-derived performance metrics is also a strength because these values represent “real-world” practice and more likely to capture realistic practice patterns than an than artificial experimental setting.

The principle limitations of this study lie in the assumptions used to build our measurement variable (CSS) and outcome variable (RT). Our CSS model does not account for NLP model error, which although uncommon, could bias the CSS from ground truth report text. Moreover, the NLP tool was validated on the complete dataset rather than the study population (n = 13,388). It is plausible that the exclusion criteria for this study may introduce additional unemeasured bias in NLP accuracy. Further, we opted to use reporting text rather than the underlying radiographic images were not referenced as a standard. Instead, our CSS presumes high fidelity translation of disease severity in the report and does not account for inter-reader variability. Nonetheless, the simplicity of the CSS provides an intuitive understanding of the interpretation process. Lastly, the generalizability of our results outside of this single academic institution is uncertain. Significant variations in reporting style, case mix, and patient demographics all likely contribute to variance in RT in other organizations and may not translate to private practice workflows.. Extrinsic factors such as time of day, day of week, and more granular clinical factors such as chief complaint are all likely important predictors of RT not included in this model. Future work should strive to integrate intrinsic (i.e. disease severity, sex, age) and extrinsic (i.e. time, date, scanner) factors to predict RT toward a more comprehensive and equitable model of radiologist efficiency.

**Conclusion:**

The CSS is an NLP-based method for analyzing lumbar MRI reports which allows *quantitative*characterization of degenerative disease severity in a large population of imaged patients. The CSS summates the cumulative severity of 18 lumbar spine components providing a global marker of LMRI study complexity which is a significant and independent predictor of radiologist reporting time. The CSS may improve existing quality metrics by allowing one to weight metrics such as the RVU by study complexity.

**Table 1:** The mean Composite Severity Score (CSS) and Interpretation and Reporting Time (RT) for the full study population and subgroups by sex and age.

**Table 2:** The relationship of CSS and RT subdivided by 10 CSS groups. Mean RT increased in a stepwise fashion (pairwise t-test) at each increment except for CSS 21-30 to 31-40 and CSS 3140 and 41-50.

**Figure 1:** Two examples of NLP analysis of archived radiology report text (1A). Using regular expression methods and the structured or semi-structured nature of LMRI spine reporting, the NLP algorithm extracted severity scores for the lumbar spine, resulting in a 6 X 3 matrix of values ranging 0-5 (normal – severe). The composite severity score is the sum of these values.

**Figure 2:** The distribution of composite severity scores (CSS), stratified by sex (2A) showing an exponential pattern. Distribution of CSS for men and women differed (K-S test p<.001). A large portion of both groups including 1797/6088 (27.87%) men, 2679/7300 (36.69%) women had CSS = 0 (normal). The proportions of the sex distributions are compared in (2B) by CSS value indicating relative greater proportion of normal and low CSS for women (0-5) and relative greater proportion of higher CSS (10-40) in men.

**Figure 3:** Distribution of Composite Severity Score (CSS) showed a moderate positive correlation between age and disease severity (Pearson’s R = 0.44, p <0.001) (3A). The distribution of CSS by age group (3B) shows greater proportion of higher CSS in older patients.

**Figure 4**: CSS was a significant predictor of RT in univariate least squares linear regression (R^2^ = 0.063, F_(1, 13386)_ = 905.6, p <0.001). Mean values for each CSS are plotted with vertical lines indicating standard error. Red dashed line shows the log-transformed linear relationship estimated by the regression model.

**Figure 5:** The relationship of CSS group to RT shown as box-and whisker plots. The box delineates the median and interquartile range. Individual CSS-RT relationship is superimposed as a color-matched dot within each CSS group. Significant pairwise increase in RT are indicated by *p<0.05, **p<0.01, ***p<0.001.

**Figure 6:** Individual radiologist practice patterns: The relationship of mean CSS and mean RT for each radiologist included in this study (n = 152), excluding 5% upper and lower tail outliers. Each point represents an individual radiologist and point size is proportional to cumulative volume of LMRI for that individual. The volume quintiles are indicated by the color scale. Vertical and horizontal lines represent median RT and CSS respectively. There was a significant positive correlation between mean CSS and mean RT (R = 0.26, p = 0.002)

**Figure 7:** The relationship of individual radiologist LMRI volume and RT, color-coding represents LMRI volume by qunitile (purple = 1^st^…yellow = 5^th^). Part A shows mean RT as a function of radiologist volume, indicating a significant negative relationship (R = -0.35, p <0.001). Vertical and horizontal lines indicate median LMRI volume (by radiologist) and median RT, respectively. Between group differences for quintiles are shown as box and whisker plots (7B); between group differences are indicated by *p<0.05, ***p<0.001.

Automatic citation updates are disabled. To see the bibliography, click Refresh in the Zotero tab.
